# Lesion-based indicators predict long-term outcomes of pheochromocytoma and paraganglioma– SIZEPASS

**DOI:** 10.3389/fendo.2023.1235243

**Published:** 2023-08-04

**Authors:** Helena Hanschell, Salvador Diaz-Cano, Alfredo Blanes, Nadia Talat, Gabriele Galatá, Simon Aylwin, Klaus Martin Schulte

**Affiliations:** ^1^ Department of Endocrine Surgery, Division of Surgery, King’s College Hospital Foundation Trust, London, United Kingdom; ^2^ Reader in Cellular and Molecular Pathology (Division of Cancer Studies), King’s Health Partners, London, United Kingdom; ^3^ Department of Pathology, University Hospital of Malaga, Malaga, Spain; ^4^ Department of Endocrinology, Division of Medicine, King’s College Hospital Foundation Trust, London, United Kingdom; ^5^ Department of Surgery, School of Medicine and Psychology, College of Health and Medicine, Australian National University, Canberra, ACT, Australia

**Keywords:** adrenal, extra-adrenal, paraganglioma, pheochromocytoma, PPGL, adrenal medulla, prediction PASS score, biomarker

## Abstract

**Aim:**

We seek a simple and reliable tool to predict malignant behavior of pheochromocytoma and paraganglioma (PPGL).

**Methods:**

This single-center prospective cohort study assessed size of primary PPGLs on preoperative cross-sectional imaging and prospectively scored specimens using the Pheochromocytoma of the Adrenal Gland Scaled Score (PASS). Multiplication of PASS points with maximum lesion diameter (in mm) yielded the SIZEPASS criterion. Local recurrence, metastasis or death from disease were surrogates defining malignancy.

**Results:**

76 consecutive PPGL patients, whereof 58 with pheochromocytoma and 51 female, were diagnosed at a mean age of 52.0 ± 15.2 years. 11 lesions (14.5%) exhibited malignant features at a median follow-up (FU) of 49 months (range 4-172 mo). Median FU of the remaining cohort was 139 months (range 120-226 mo). SIZEPASS classified malignancy with an area under the curve (AUC) of 0.97 (95%CI 0.93-1.01; p<0.0001). Across PPGL, SIZEPASS >1000 outperformed all known predictors of malignancy, with sensitivity 91%, specificity 94%, and accuracy 93%, and an odds ratio of 72 fold (95%CI 9-571; P<0.001). It retained an accuracy >90% in cohorts defined by location (adrenal, extra-adrenal) or mutation status.

**Conclusions:**

The SIZEPASS>1000 criterion is a lesion-based, clinically available, simple and effective tool to predict malignant behavior of PPGLs independently of age, sex, location or mutation status.

## Introduction

Chromaffin and neural progenitor cells share many features (1) and cross-differentiate from the same progenitors (2). Tumors derived from chromaffin progenitor cells in the adrenal medulla (3), or from autonomic paraganglia of the sympathetic or parasympathetic nervous system, define the group of non-epithelial neuroendocrine tumors (4). However, nestin-positive progenitor cells or Sox-10 deplete-initiated chromaffin cells may escape physiological controls to form tumors (5). These neoplasms are commonly referred to as paragangliomas (PGL), the special and more prevalent case of adrenal tumors identified as adrenal paraganglioma or pheochromocytoma (PCC), whilst all other lesions are identified as extra-adrenal paragangliomas (6). The joint cohort of adrenal and extra-adrenal lesions is identified as pheochromocytoma paraganglioma (PPGL).

PPGL cause significant harm and necessitate surgical resection. Major adverse events are driven by excessive catecholamine secretion. They include cardiovascular events such as arterial hypertension with hypertensive crisis, arrhythmia or cardiomyopathy to the point of cardiac failure (7), stroke, kidney damage, and derailed glucose homeostasis. Space-occupying effects of primary lesions are uncommon, but malignant invasion of major organs or vessels, local recurrence following surgical removal, distant metastasis or ongoing catecholamine excess, may eventually result in death.

Little is known how to select at-risk patients for more stringent FU or adjuvant therapy. An upfront diagnosis of malignancy would be of paramount importance. However, in absence of clear-cut histopathological features, the WHO abandoned classification of PGLs as benign and malignant. Instead, any PPGL is considered to have malignant potential (6). This approach is academically justified, yet it fails to satisfy core demands of clinical care. Clinicians need to provide the patient with a reasonably confident outlook on their prognosis and the expected life impact of their condition, whilst reliable risk assessment would help steer follow-up and eventual pre-emptive treatment interventions.

There is a quest for clinical, histopathological or genetic markers predicting malignant behavior (8). Critical insight can be gleaned from a large, retrospective multicenter study comprising 1127 patients with PPGLs, genotyping, and long-term follow-up (9). Whilst 70% of recurrences occurs within the first decade, 17.7% of lesions occur only after 15 years (9). The nature of driver mutations, or their absence, defines three clusters with varying degrees of probability of being malignant (9). Sporadic PPGLs recur at a rate of 14.7%, similar to 14.9% with mutations in kinase pathways, whilst 47.5% of lesion with mutations in hypoxia pathways recurred (9). In sporadic PPGLs, critical features associated with malignancy are a noradrenergic pattern of hormone secretion, tumor size greater than 5cm, and extra-adrenal location, whilst age and sex had no importance (9).

Many former studies are limited by follow-up. Some scores are exclusively based on clinical criteria, e.g. ASES (10), or histopathological criteria, e.g. PASS (11, 12), whilst others combine features from both categories, e.g. GAPP (13, 14), mGAPP, COPPS (15), or SGAP (16–18). Of these, the Composite Pheochromocytoma/paraganglioma Prognostic Score (COPPS), including tumor size, necrosis, and vascular invasion as well as loss of immunohistochemical staining for PS100 and SDHB, stands out with a sensitivity of 100% and a specificity of 94.7%, whilst the PASS score performed with a sensitivity of merely 66.7% in the same cohort (15). The bulk of work support histopathological criteria identifiable from routinely stained samples and the age-old established malignancy predictor lesion size as key independent predictors of malignant behavior.

Promoting health equity and practice relevance of academic progress supports extending exploration beyond high-end biomarkers from advanced sequencing or subspecialist immunohistochemistry to more accessible information from common clinical-diagnostic pathways such as imaging or histopathology with conventional staining. Mathematical modelling supposes that scores derived from adding score points or dependent on the upfront categorical classification of individual variables are vulnerable to bias at threshold definitions. We therefore investigate the predictive value of a score derived from multiplication, rather than addition, of histopathological scoring and lesion size.

## Materials and methods

### Patients

We refer to definitions as per the World Health Organization. Chromaffin tumors of the second sympathetic of parasympathetic neurons are termed paraganglioma (PGL). Those tumors located in the adrenal are identified as “adrenal PGL” or pheochromocytoma (PCC), whilst all other PGLs are identified as “extra-adrenal PGL”; together, adrenal and extra-adrenal PPGLs are identified as PPGLs.

A series of consecutive patients with PPGL (n=78) were treated in a single tertiary adrenal referral center between 2004 and 2013. We excluded two patients with an initial diagnosis of multifocal PPGLs, because the origin of any recurrence could not have been ascertained from any specific lesion and hence its size or PASS. Patients underwent pre- and post-operative scrutiny in King’s Adrenal Multidisciplinary Team Meeting (MDT) with complete retention and discussion of data on clinical and biochemical presentation, treatment modality, histology and outcomes. The MDT comprises specialist input from endocrinology, endocrine surgery, radiology, nuclear medicine, histopathology, clinical chemistry and, where appropriate, clinical oncology and genetics. Comprehensive MDT reports and King’s online Electronic Patients Record (EPR) record relevant data and follow-up. For this study, all data were retained in a deanonymized state only, and no data enabling patient recognition are presented in this study. The study was assessed by the United Kingdom’s Integrated Research Application System (IRAS), IRAS Project ID 329934.

### Evaluation of size

We observed significant differences between the gross size dimensions of the histopathological specimens and the size of PPGLs on preoperative cross-sectional imaging (unpublished data), most likely due to volume losses, when arteries are clamped first, and volume gains, when lesion veins are clamped first. We therefore assessed lesion size on preoperative computed tomograms (CT) or magnetic resonance tomograms (MRI), at least one of which was available in all cases. Two independent examiners (HH, KMS) assessed maximal lesion diameter on transverse, coronal, and transverse reconstructions. The statistical analysis included only the largest diameter for each lesion.

### Evaluation of histopathological criteria and mutations

Specimens were generously sampled. Tumors less than 5cm were wholly embedded. We embedded 2-3 blocks per cm length of maximum dimension for lesions larger than 5cm. All hematoxylin and eosin-stained (H&E) slides from every patient were reviewed for morphologic assessment. The Pheochromocytoma of the Adrenal gland Scaled Score (PASS) was evaluated as described and applied using the criteria initially proposed (11). All cases were independently reviewed by two pathologists (SDC, AB) commanding expertise in the histopathology of endocrine tumors and who were blinded to clinical outcomes (recurrence, metastasis). In case of disagreement in risk stratification, lesions were discussed during simultaneous inspection before final categorization. Inter-observer agreement was assessed using κ statistics; the strength of the former was evaluated with criteria previously described by Landis and Koch (19). Mutation testing was performed according to guidelines. Panels varied over time.

### Definition of outcome events

All patients were followed up for at least 120 months (10 years) after diagnosis or until death from PGL or other causes. Lesion behavior was defined as “malignant” if either of the following criteria was met: a) at the time of diagnosis: imaging evidence of locoregional (lymph nodal metastasis) or distant metastasis or histological evidence of tumor cells at sites where chromaffin tissue is not usually present (e.g. lymph nodes, liver, or bone) (20) b) locoregional recurrence during follow-up (i.e. imaging or histologic proof of PGL tissue at the site of prior resection or in neighboring lymph nodes) after initial complete tumor eradication (documented by negative biochemical and imaging tests) (21) or c) metastasis during follow-up. Of note, the definition of recurrence or metastasis excluded the occurrence of new PPGLs affecting the contralateral adrenal or ectopic chromaffin tissue in patients with known familial PCCs. Time to recurrence in months was counted between initial surgery and documentation of recurrence. Outcome analysis for recurrence-free survival (RFS) was defined as either death due to disease and/or recurrence/persistence of disease at any time.

### Statistical evaluation

Means between groups were compared using Student’s t test. Univariate survival analysis was performed using Kaplan-Meier analysis. Performance of the classification model at all classification thresholds was analyzed using receiver operating characteristic curves (ROC curves). Areas under the curve (AUC) values of 0.8-0.9 were defined as excellent; AUC values of 0.9-1.0 were defined as outstanding (22). For all tests, results with two-sided analysis meeting a threshold of p<0.05 were considered statistically significant. Statistical analyses were performed using SPPS statistics software version 27.0 (IBM). Evaluation of criteria for their performance as diagnostic test was performed using the MedCalc Software Ltd. Diagnostic test evaluation calculator (Version 22.009; accessed July 5, 2023), https://www.medcalc.org/calc/diagnostic_test.php. Data are corrected for prevalence of malignant behavior in the analyzed cohort, i.e. 11/76 or 14.5% for the PPGL cohort or prevalence in specific sub-cohorts analyzed.

## Results

The cohort of n= 76 individuals, mean age 52.0 ± 15.2 years, comprised 51 female patients (67.1%). During the observation period, a malignant phenotype was identified in 11 patients, 6 in female patients, with no sex correlation (Pearson 2.73 and Fisher exact test at p=0.1). It manifested as locoregional recurrence or metastasis at a median follow-up of 49 months (range 4-172 mo). The remaining cohort was followed for a median of 141 months (mean 152 ± 30 mo; range 120-226 mo) or until death.

Genetic testing was performed in 35/76 patients (46.1%). It identified driver mutations in the hypoxia pathway in 15/35 (42.9%) and kinase pathways in 4/35(11.4%). Follow-up for patients with sporadic PPGLS was a median of 132 months (mean 136 ± 42; range 30-226), with a median of 90 months (mean 100 ± 68; range 10-210) in the group of hereditary PPGLs.

**Figure 1:**
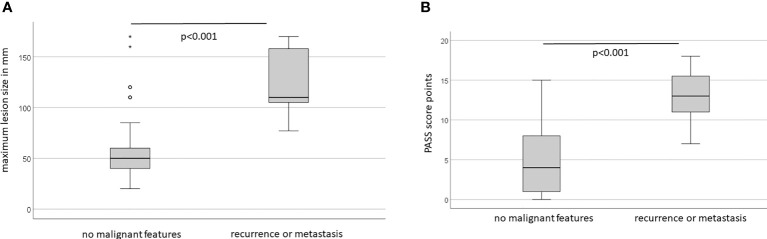
**(A)** Lesion size varies with long-term clinical behaviour. **(B)** PASS score points vary with long-term clinical behaviour.

**Figure 2:**
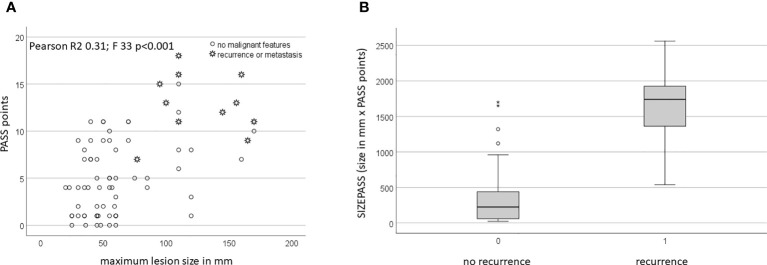
**(A)** PASS scores vary with size. **(B)** SIZEPASS varies with long-term biological behaviour.

Lesion size, defined as largest diameter on cross-sectional imaging in mm, did not significantly vary with age or sex (female 67 ± 41 vs 71 ± 40; p=0.7). Malignant lesions were larger than benign lesions, 127 ± 40mm vs 59 ± 31mm (ANOVA F=44.2; p<0.001) (**Figure 1A**). Used as continuous parameter, lesion size was a powerful classifier with an AUC of 0.92 (95%CI 0.86-0.98; p<0.0001). PASS points did not significantly vary with age or sex (female 5.2 ± 4.4 vs male 7.6 ± 5.0; p=0.053). Average PASS was significantly higher in malignancy (12.8 ± 3.3 vs 4.8 ± 3.8; F=42 p<0.001) (**Figure 1B**). Used as continuous variable, PASS showed excellent performance with an AUC of 0.94 (95%CI 0.87-1.00; p<0.0001) with an overall model quality of 0.87. However, PASS failed to exhibit a clear bimodal distribution when plotted for malignancy status (**Figure 2A**).

Jointly, PASS and size identified two fairly distinct cohorts of small tumors with low PASS and large tumors with high PASS (**Figure 2A**). Size and PASS showed a moderate, but significant correlation (Pearson coefficient R 0.56; p<0.01), yet in an overall weak model (R^2^ 0.31 with adjusted R^2^ of 0.30), with similar outcomes in the benign cohort (Pearson coefficient 0.34 with p<0.005), but not the malignant cohort (Pearson coefficient 0.02 with p=0.95). Accordingly, linear regression analysis showed no dependence of PASS on size. Whilst the addition of PASS points and size did not add value to models (data not shown), the product resulting from multiplication of PASS points with size (in mm), defined as SIZEPASS, strongly discriminated malignant from benign lesions (328 ± 373 vs 1627 ± 526; p<0.001) (**Figure 2B**).

**Figure 3:**
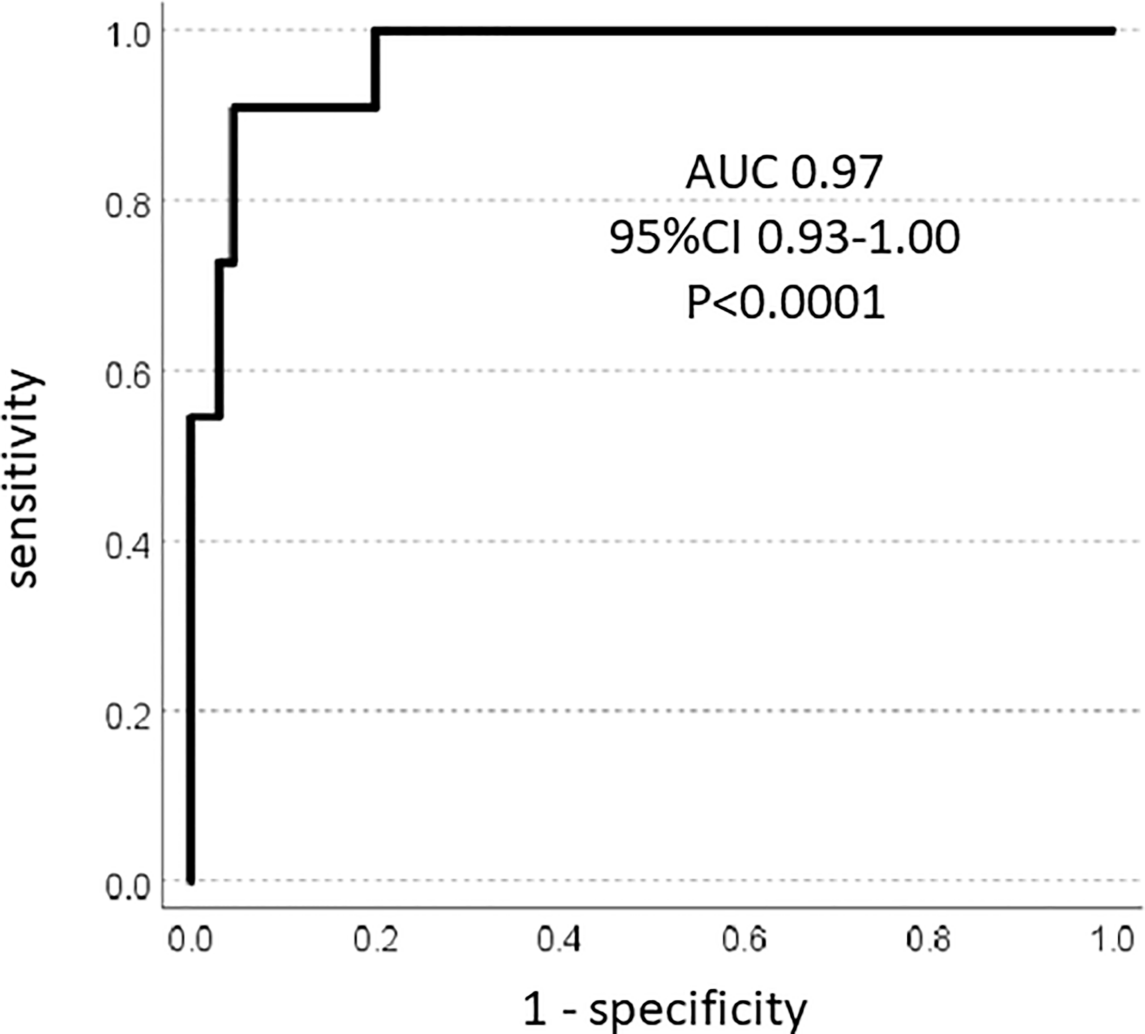
ROC analysis: SIZEPASS classifies malignancy.

**Figure 4:**
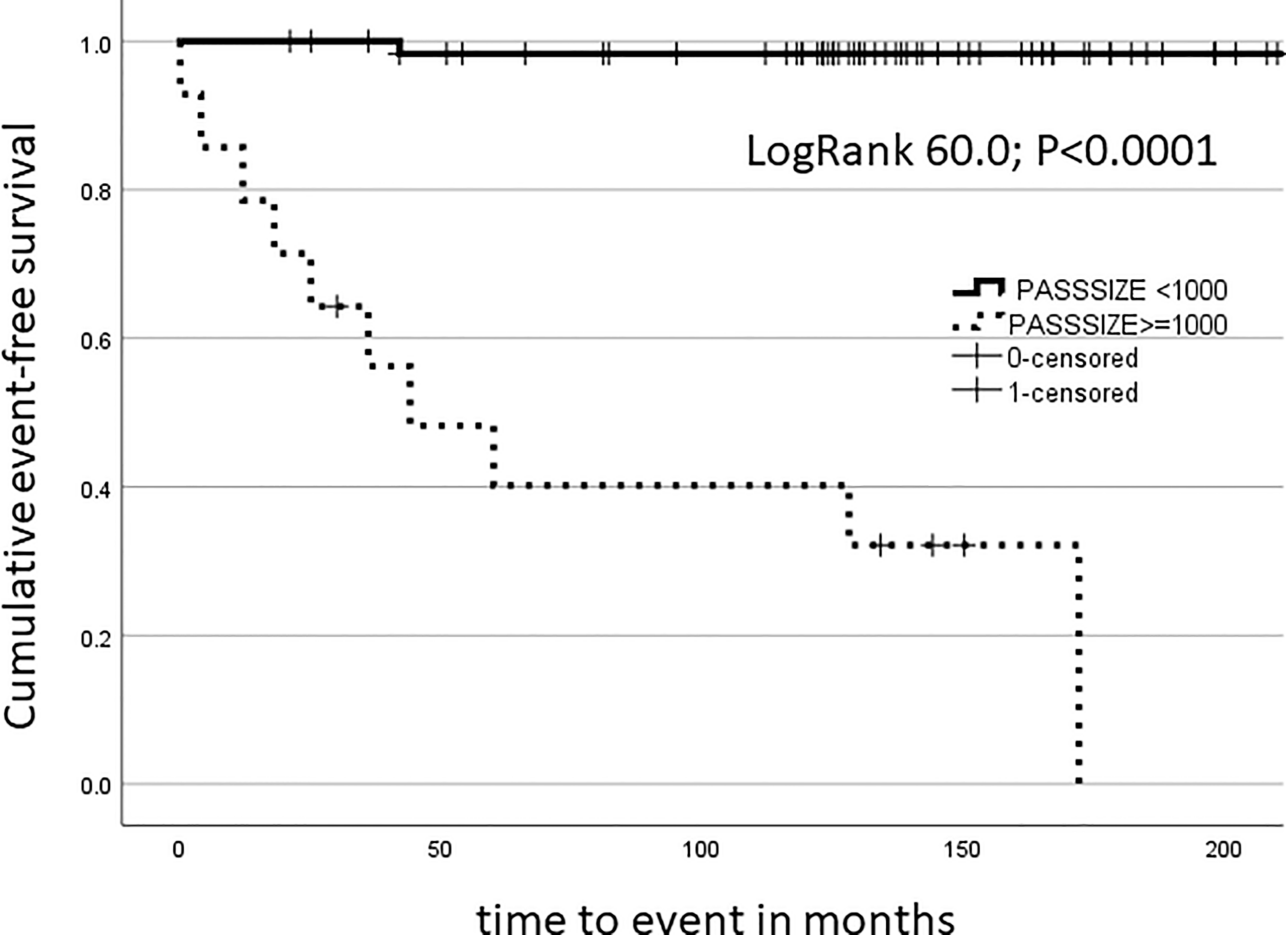
Kaplan-Meier analysis: SIZEPASS1000 discriminates cohorts for long-term malignancy outcomes.

As a continuous parameter, SIZEPASS showed excellent performance in univariate binomial logistic regression analysis with ExpB 1.50 (95%CI 1.22-1.83; p<0.001) and correctly classified 93.4% correct classification of tumours as benign or malignant. This was confirmed by ROC analysis showing an AUC of 0.97 (95%CI 0.93-1.01; p<0.0001) (**Figure 3**) and an overall model quality of 0.93. To render this a clinically useful tool, we incrementally checked thresholds for a binary SIZEPASS criterion and observed optimal performance at 1000 points yielding an AUC of 0.92 (95%CI 0.82-1.03; p<0.0001), which showed excellent performance in Kaplan-Meier analysis (**Figure 4**).

At the threshold of 1000, the goodness of fit analysis by Pearson’s χ^2^ test showed a value of 45.0 (1 degree of freedom (df), p<0.001) with a likelihood ratio of 35.9 (1df; p<0.001) and Fisher’s exact test at 44.4 (p<0.001). Calculation of predictive power of size and PASS points at various thresholds and comparison with the SIZEPASS1000 criterion showed superior performance for the combined criterion over various size criteria (**Table 1**).

**Table 1:** A binary SIZEPASS1000 criterion outperforms size and PASS for the classification of malignancy of PPGLs.

criterion	sensitivity	specificity	PPV	NPV	Accuracy
SIZEPASS1000	90.9%	**93.9%**	**73.3%**	**98.5%**	**93.4%**
PASS ≥10	81.8%	86.2%	50.0%	96.6%	85.5%
size ≥60mm	**100%**	66.2%	33.3%	66.2%	71.1%
size ≥70mm	**100%**	76.9%	42.3%	76.9%	80.3%
size ≥80mm	90.9%	83.1%	52.3%	81.8%	84.2%
size ≥90mm	90.9%	86.2%	57.9%	84.8%	86.8%
size ≥100mm	81.8%	86.2%	61.1%	83.6%	85.5%

Optimal performance is indicated in bold. Size criteria >110mm failed. Only the optimal threshold for PASS is shown.

The classification power of the SIZEPASS1000 criterion significantly exceeded that of established score systems such as PASS and size criteria which we tested at the upper published dichotomy thresholds (**Table 2**). PASS and size criteria performed worse when used at commonly employed threshold of PASS ≥4 and size ≥5cm (data not shown).

**Table 2:** Classification power of tools predicting malignant behavior in 76 patients with PPGL. ROC Analysis.

prediction tool (dichtomized variables)	AUC	95% CI lower	95% CI upper	Significance P two-sided
age >50 years	0.51	0.33	0.70	0.91
sex (male)	0.63	0.44	0.81	0.18
mutation positive (yes)	0.72	0.54	0.90	0.02
size >60 mm	0.83	0.74	0.92	<0.0001
PASS score points >6	0.82	0.73	0.92	<0.0001
SIZEPASS>1000	0.92	0.82	1.03	<0.0001

Malignant behavior of pheochromocytoma or paraganglioma (PPGL) was identified as metastatic disease at any time, recurrence or death from disease. Mutation status was positive if germline mutations in hypoxia pathways were identified and negative if no mutation was identified or patients had not been tested. Predictors were dichtomized according to prior use (size and PASS) or as identified from this cohort (SIZEPASS). Results from receiver operating curve analysis (ROC) is presented as area under the curve (AUC) with its 95% confidence interval (CI).

In Cox regression survival analysis, SIZEPASS outperformed size, PASS, sex or mutation status in uni-variate analysis and persisted in multivariate analysis. The excellent performance observed when using continuous variables, size (odds 1.030 95%CI 1.017-1.044; p<0.001) and PASS (odds 1.7 95%CI 1.4-2.2; p<0.001) (for full data see **Supplementary Table 1**), disappeared after dichotomization even at the upper end of widely used thresholds, i.e. size >60mm and >6 PASS score points. In multivariate analysis, only mutation and SIZEPASS1000 criterion persisted (**Table 3**).

**Table 3:** Cox survival regression Outcome: metastasis at any time or recurrent disease or death from disease.

UNIVARIATE ANALYSIS					
predictor (dichotomized variables)	χ^2^	*P*	odds ratio	95% CI lower	95% CI upper
age >50 years	0.1	*0.8*	1.2 P=0.7	0.4	4.0
sex (male>female)	3.4	*0.08*	3.0 P=0.08	0.9	9.8
size in mm >60 mm	17.5	*<0.001*	117.4 P=0.07	0.7	19297
mutation positive	11.0	*<0.001*	6.2 P=0.004	1.8	21.3
PASS score points >6	15.6	*<0.001*	98.4 P=0.07	0.7	14409
SIZEPASS >1000	59.6	*<0.001*	72.4 P<0.001	9.2	571
MULTIVARIATE ANALYSIS Factors entered: age>50, sex, and each other single parameter below					
predictor (dichotomized variables)	χ^2^	*P*	odds ratio	95%CI lower	95% CI upper
size >60 mm sex	21.5	*<0.001*	4x10^5^ ^P=0.926^	–	–
mutation positive	12.8	*<0.005*	5.8 P=0.003	1.5	22.3
PASS score points >6	37.5	*<0.001*	2.7x10^5^ ^P=0.926^	–	–
SIZEPASS >1000	69.3	*<0.001*	72.4 P<0.001	9.2	571.4

When mutations status (yes/no) and SIZEPASS>1000 criterion were co-entered into multivariate COX-regression analysis, only SIZEPASS1000 survived (odds ratio 54.7; 95%CI: 6.7-449.7; P<0.001). Equally, when PASS score points >6 and SIZEPASS>1000 criterion were co-entered into multivariate COX-regression analysis, only SIZEPASS1000 survived (odds ratio 23.8; 95%CI: 3.0-188.1; P=0.003).

The SIZEPASS1000 criterion exhibited excellent performance when evaluated as diagnostic test. An accuracy ≥90%, was observed across cohorts with different mutation status (**Table 4**). This included a mutation-agnostic approach (all PPGL) or cohorts of PPGL, where either no mutation had been identified on testing (n=14) or where the Adrenal MDT had not identified a clinical indication for testing (n=42).

**Table 4:** The binary SIZEPASS1000 criterion performs across mutation status.

cohort	sensitivity 95%CI	specificity 95%CI	PPV 95%CI	NPV 95%CI	accuracy 95%CI
all PPGL (n=76)	91% 59-100%	94% 85-98%	71% 42-92%	98% 91-100%	93% 85-98%
mutation-negative/untested (n=56)	100% 40-100%	94% 84-99%	57% 18-90%	100% 93-100%	95% 85-99%
hypoxia pathway mutation (n=13)	83% 36-100%	100% 59-100%	83% 48-100%	88% 47-100%	92% 64-100%
any mutation (n=20)	86% 42-100%	92% 64-100%	86% 42-100%	92% 64-100%	90% 68-99%

PPV, positive predictive value; NPV, negative predictive value; PPGL, pheochromocytoma and extra-adrenal paraganglioma; PGL, paraganglioma; 95%CI = 95% confidence interval assessed via MedCalc Software Ltd. Diagnostic test evaluation calculator https://www.medcalc.org/calc/diagnostic_test.php (Version 22.009; accessed July 5, 2023); data are corrected for prevalence of malignant behavior in the sub-cohort analyzed.

Comparison of size, PASS and SIZEPASS criteria in cross table analysis confirmed the superiority of the SIZEPASS1000 criterion, particularly with regard to higher specificity and positive prediction (**Table 5**).

**Table 5:** Comparative evaluation of prediction tools for malignant behavior in 76 patients with PPGL.

Prediction tool	χ^2^ *P*	odds ratio 95% CI	sens. %	spec. %	PPV %	NPV %	accuracy %
mutation status positive	9.2 0.002	7.0 1.8-27.6	63.7	80.0	35.1	92.8	77.6
size >60 mm	16.8 <0.001	3.0 2.1-4.2	100	66.2	33.4	100	71.0
PASS score points >6	15.9 <0.001	2.8 2.0-3.9	100	64.6	32.4	100	69.8
SIZEPASS >1000	45.0 <0.001	152.5 15.4-1507	90.9	93.9	71.5	98.4	93.4

Malignant behavior was identified as metastatic disease at any time, recurrence or death from disease. Predictors were dichotomized according to prior use (size and PASS) or as identified from this cohort (SIZEPASS). Cross table correlations are presented as Pearson χ^2^ with its two-sided significance level P. Mutation status was positive if any germline mutations were identified and negative if no mutation was identified or patients had not been tested for want of clinical indication.

## Discussion

This study identifies the excellent performance of the SIZEPASS1000 criterion to distinguish benign from malignant clinical behavior of PPGL. The criterion is derived by multiplying PASS points with the maximum lesion diameter taken from preoperative cross-sectional radiological studies. The approach employs criteria previously recognized to predict malignant behavior of PPGLs, i.e. size (9, 23) and PASS score (11, 24).

Common opinion has long held a PPGL size of 5-6 cm or above as a clinical indicator of malignancy (25). This is confirmed in the largest to-date dataset of PPGLs, where size >5 cm predicted malignancy (HR 1.67; CI, 1.040-2.668; P=0.034) and persisted as independent predictor of recurrent disease in multivariable Cox regression (9). However, numerous authors identified that lesions of “intermediate size”, defined as a maximum diameter of 4-10 cm, are not necessarily malignant (23, 26). Size estimates of pathological specimens suffer from inconsistencies in defining the largest diameter of the specimen, and perhaps more importantly from the impact of operation technique on the volume of the commonly heavily vascularized lesion. Vein first approaches engorge the sample, whilst early arterial clamping may permit effective size reduction of such tumors (HH and KMS, unpublished data). We have therefore employed pre-operative cross-sectional imaging, which was available in all cases, and have ensured exact measurement by two independent observers (HH and KMS).

A multitude of studies have validated the PASS. Early investigations identified high classification power of a PASS: when used as continuous parameter it achieved an AUC of 0.90 (24). A critical problem of PASS, which employs a panel of distinct criteria, is significant inter-observer and intra-observer variation (27). Variation is not just reduced to the apparent problem of intra-tumoral heterogeneity, but also to varying interpretation of findings in any given sample (27). All PASS scores in the current presentation were produced by two pathologists (SDC, AB) before patients entered long-term follow-up (2013-2023), thereby limiting inter-observer variation and granting a truly prospective assessment of specimens. It should also be said, that the consistently good performance of PASS across a multitude of cohorts identified in a meta-analysis including more than 900 patients (28), argues against the claim that observer bias would acutely detract from the predictive value of the PASS score.

Problems arise, however, when defining any specific threshold value above which the PASS would identify malignancy. Most authors have proposed cut-off values of ≥4 (11, 23, 26, 29–33) or ≥6 points (25, 34), whilst other authors found that neither cut-off point was valid (35, 36). Notwithstanding prior criticism of intra- and inter-observer variation, an excellent meta-analysis from the Karolinska Institute including 28 studies has confirmed the classification power of PASS ≥4 in a cohort of 848 adrenal PGL and 56 extra-adrenal PGL (28).

The key problem with cut-off values is the requirement of decision-making about the relative impact of a particular expression of any variable early in the analytical process. An analogous problem occurs when values are added, favoring parameters with inherently higher numerical values. Both problems can be circumvented by multiplication (or division). We have here used the product of two well-established lesion-based predictors of malignancy, namely size and PASS, which we confirm to be highly different between benign and malignant lesions (**Figure 1**). However, the degree of scatter makes the selection of any particular cut-off point for either variable problematic (**Figure 2A**), whilst the product of these two criteria shows better discrimination (**Figure 2B**). These benefits consistently extend into classification analysis employing receiver operating curves, where the individual criteria perform highly before, but not after, dichotomization. In contrast, the size x PASS product loses little traction and exhibits excellent performance with an AUC above 0.8 (**Figure 3**). This again translates into higher predictive power and accuracy compared to the individual criteria (**Tables 1**, **2**, **4**, **5**). These observations compare large-volume meta-analytical data (**Table 6**), where the SIZEPASS1000 criterion outperforms both PASS and GAPP scores (28). We note that the GAPP score (13, 14) underperforms in meta-analysis, an observation confirmed in later publications (9, 32, 37).

**Table 6:** The binary SIZEPASS1000 criterion outperforms PASS≥4cm and GAPP≥3 for the prediction of malignancy across PPGLs, as well as in adrenal and extra-adrenal PGL cohorts.

criterion	sensitivity 95% CI	specificity 95% CI	PPV 95% CI	NPV 95% CI	accuracy 95% CI
PPGLs n=76					
SIZEPASS1000	91% 59-100%	94% 85-98%	71% 42-92%	98% 91-100%	93% 85-98%
PASS≥4 n=851*	97%	68%	33%	99%	72%
GAPP≥3 n=210*	75%	99%	12%	78%	77%
adrenal PGLs n=58 (PCC)					
SIZEPASS1000 n=58	100% 40-100%	93% 82-98%	50% 16-84%	100% 93-100%	93% 83-98%
PASS≥4 n=848*	97%	68%	31%	99%	72%
GAPP≥3 n=199*	50%	80%	5%	99%	79%
extra-adrenal PGLs n=18					
SIZEPASS1000	85.7% 42-100%	86% 72-100%	100% 54-100%	92% 62-100%	94% 73-100%
PASS≥4 n=56*	100%	72%	62%	100%	81%
GAPP≥3 n=51*	100%	68%	29%	100%	71%

PPV, positive predictive value; NPV, negative predictive value; PPGL, pheochromocytoma and extra-adrenal parapanglioma; PGL, paraganglioma; *values calculated from cases with available data as presented in Stenmann et al., 2019 (28); MedCalc Software Ltd. Diagnostic test evaluation calculator. https://www.medcalc.org/calc/diagnostic_test.php (Version 22.009; accessed July 5, 2023), data are corrected for prevalence of malignant behavior in the cohort analyzed.

Our data also demonstrate the SIZEPASS1000 criterion to outperform mutation status as risk predictor (**Table 3**). It keeps its predictive power regardless of mutation status (**Table 4**). SIZEPASS1000 and mutation analysis occupy complementary functions: a SIZEPASS >1000 indicates a very high risk of malignant behavior, prompting dedicated diagnostic interventions. It may help with complex considerations regarding adjuvant therapy choices. A positive mutation status informs the need to monitor for formation of secondary PPGLs and eventual care needs for relatives.

In PPGL, the evaluation of outcomes predictors is limited by the extreme delay of locoregional recurrence or metastases. The to-date largest collection of data comprises 298 patients with sporadic PGL: 63% of locoregional recurrences and 69% of metastases were observed within the first decade of follow-up, with saturation reaching 81% and ~100% at 15 years of follow-up (9). Except for those dying of other causes, all of our patients were followed up for at least 10 years, with a median length of follow-up of 11.7 years. The observation supposes that at least 70% of all expected local recurrence and nearly all metastatic events would have occurred within the observation period in our cohort. Whilst still an imperfect scenario, our data offer sufficient follow-up to inform a robust analysis.

Our data do not afford comparison with alternative scoring approaches, such as the ASES score based on mere clinical observations, yet its performance is limited with an AUC of only 0.74 (10, 38–40). On the other hand, the COPPS score appears to offer excellent performance, yet it is mitigated by the need for specialized immunohistochemistry (15).

No score based will ever substitute for a clear-cut definition of malignancy such as histopathology delivers for most, though not all, solid tumors. We agree that a definition of malignancy based on “absolute” criteria would be preferable, yet those are elusive. A comprehensive discussion of individual criteria and select scores has been provided in the 2022 overview of the classification of paragangliomas and pheochromocytomas (6). Mete and colleagues agree that the PASS is the best performing system so far, yet commend caution regarding its use, as indeed for any other scoring system: “*Therefore, the 2022 WHO classification of PPGLs does not endorse any of these systems, but at the same time does not discourage their use in individual practices. It is stressed that scoring systems may be used optimally in relation to the results of molecular analyses [86, 87], as well as other clinical and biochemical (e.g., mature or immature secretory phenotype) risk factors.*”(6).

Our proposed novel criterion for PASS, the SIZEPASS1000 criterion, benefits patients and care providers alike. The SIZEPASS1000 criterion is solely based on two objectively assessable lesion-related criteria, i.e. size and histopathological appearance which can be recognized in basic H&E stains. This prevents problems arising from consideration of ever new immunohistochemical markers, and obviates the need for assumptions about genetic versus allegedly sporadic manifestations. The latter is particularly problematic as a growing number of novel genetic lesions are identified to drive or at least interact with the biological behavior of PGLs. During the three decades since the mapping of the MEN2 locus to chromosome 10 (41), multi-omics definition of principal driver lesions (42), recognition of key aberrations in DNA repair altering lesion evolution (43), and a growing approach including alterations in mitochondrial genes (44, 45), have created an increasingly complex and complete, yet imperfect, picture of genetic causation. When the majority of PPGLs was considered sporadic in 1990, this group has shrunk to merely 30-50% at present (9, 46); and it may shrink further. Assessment of a majority of currently available genetic targets has considerable complexity, and the ever-novel immuno-histochemical tests are by no means cheap. Affluence or eligibility criteria defined by the territorial commissioning systems could hence become significant factors in the recognition of potential malignancy in PGL, as in any other genetically driven conditions. This is not helped by the observation that genetic variation is unequally represented across ancestry groups (47, 48). The growing genetic knowledge base in PGL is undoubtedly highly meritorious, yet does it drive complexity and cost to contribute to health inequity in underrepresented populations (49). Our data (**Tables 3**, **5**, results section) identify that the SIZEPASS1000 criterion outperforms mutation status in our cohort, when entered as competitive co-factor into multifactorial Cox survival regression. This finding further corroborates our claim that the SIZEPASS1000 criterion may be used in any cohort, and without prior knowledge of mutations status.

From a practical point of view, patients are keen to learn about the real-life impact of their newly diagnosed PGL. The SIZEPASS1000 criterion offers a simple, lesion- based and outstandingly performing tool to answer their questions. It can be determined without access to complex, expensive or cumbersome genetic or immune-histochemical tests. It offers answers in all such scenarios where family history is elusive, and genetic tests unavailable, culturally unacceptable, ambiguous, or not sufficiently explored for impact because they are rare, with insufficient long-term studies or knowledge of cooperating genetic factors. In addition, the growing variety of PGL driver genes often lacks sizable long-term studies robustly connecting them to their clinical phenotype, particularly for any late sequel such as recurrence.

We hope that the universally accessible nature of SIZEPASS score compounds, namely PASS and lesions size from cross-sectional imaging, will enable validation of our findings in other cohorts. Once that is eventually achieved, SIZEPASS1000 can improve cohort stratification in much needed randomized future trials and may help to select patients for adjuvant therapies such as endoradiotherapy.

The SIZEPASS1000 criterion outperforms current histology-based or size-based approaches, adding to the armamentarium of prognostication of a rare, but important, endocrine tumor. It does not replace current efforts aimed at better understanding the nosology of PGLs, but may have value as a complement to current and future clinical practice.

## Data availability statement

The raw data supporting the conclusions of this article will be made available by the authors, without undue reservation.

## Ethics statement

The studies involving human participants were reviewed and approved by United Kingdom’s Integrated Research Application System (IRAS), IRAS Project ID 329934. Written informed consent for participation was not required for this study in accordance with the national legislation and the institutional requirements.

## Author contributions

HH: Investigation, Writing-review and editing. SD-C: Conceptualization, Investigation, Writing-original draft, Writing-review and editing. AB: Investigation, Writing-review and editing. NT: Investigation, Writing-review and editing, Supervision. GG: Investigation, Writing-review and editing, Supervision. SA: Writing-review and editing, Supervision. K-MS: Conceptualization, Investigation, Writing-original draft, Writing-review and editing.
