# Supplementary material for: Lesion-based indicators predict long-term outcomes of pheochromocytoma and paraganglioma– SIZEPASS
Source: Front Endocrinol (Lausanne). 2023 Aug 4;14:1235243. doi: 10.3389/fendo.2023.1235243 (PMC10436571; doi:10.3389/fendo.2023.1235243)
Supplement: Supplementary file 1 [file Table_1.pdf]

| Supplemental Table 1: Cox survival regression<br>Outcome: metastasis at any time or recurrent disease or death from disease | | | | | |
|---|---|---|---|---|---|
| UNIVARIATE ANALYSIS | | | | | |
| prediction tool<br>(**continuous variables**) | $\chi^2$ | *P* | odds ratio | 95%CI lower | 95% CI upper |
| age | 0.2 | *0.2* | 1.0<br>P=0.6 | 1.0 | 1.0 |
| gender (male>female) | 3.4 | *0.08* | 3.0 | 0.9 | 9.8 |
| size in mm | 32.5 | *<0.001* | 1.030<br>P<0.001 | 1.017 | 1.044 |
| mutation status positive for hypoxia pathway (yes>no) | | | | | |
| PASS score points | 36.7 | *<0.001* | 1.7<br>P<0.001 | 1.4 | 2.2 |
| SIZEPASS | 68.4 | *<0.001* | 1.004<br>P<0.001 | 1.002 | 1.005 |
| prediction tool<br>(**dichtomized variables**) | | | | | |
| age >50 years | 0.1 | *0.8* | 1.2<br>P=0.7 | 0.4 | 4.0 |
| gender (male>female) | 3.4 | *0.08* | 3.0<br>P=0.08 | 0.9 | 9.8 |
| size in mm >60 mm | 17.5 | *<0.001* | 117.4<br>P=0.07 | 0.7 | 19297 |
| PASS score points ≥6 | 15.6 | *<0.001* | 98.4<br>P=0.07 | 0.7 | 14409 |
| SIZEPASS ≥1000 | 59.6 | *<0.001* | 72.4<br>P<0.001 | 9.2 | 571 |
| MULTIVARIATE ANALYSIS<br>Factors entered: age>50, gender, and each single parameter below (as continuous variable) | | | | | |
| Predictor<br>(**continuous**) | $\chi^2$ | *P* | odds ratio | 95%CI lower | 95% CI upper |
| mutation status positive for hypoxia pathway (yes>no) | | | | | |
| size in mm<br>gender | 35.1 | *<0.001* | 1.034<br>4.0 | 1.018<br>1.0 | 1.050<br>16.2 |
| PASS score points (higher>lower) | 37.5 | *<0.001* | -<br>P=0.928 | - | - |
| SIZEPASS (higher versus lower) | 59.6 | *<0.001* | 72.4<br>P<0.001 | 9.2 | 571.4 |
| Predictor (**dichotomized**) | | | | | |
| size in mm<br>gender | 21.5 | *<0.001* | $4x10^5$<br>P=0.926 | - | - |
| PASS score points (higher>lower) | 37.5 | *<0.001* | $2.7x10^5$<br>P=0.926 | - | - |
| SIZEPASS (higher versus lower) | 69.3 | *<0.001* | 72.4<br>P<0.001 | 9.2 | 571.4 |
